# Targeted Activation of Hippocampal Place Cells Drives Memory-Guided Spatial Behavior

**DOI:** 10.1016/j.cell.2020.09.061

**Published:** 2020-12-10

**Authors:** Nick T.M. Robinson, Lucie A.L. Descamps, Lloyd E. Russell, Moritz O. Buchholz, Brendan A. Bicknell, Georgy K. Antonov, Joanna Y.N. Lau, Rebecca Nutbrown, Christoph Schmidt-Hieber, Michael Häusser

**Affiliations:** 1 Wolfson Institute for Biomedical Research and Department of Neuroscience, Physiology and Pharmacology, University College London, London WC1E 6BT, UK

**Keywords:** place cell, hippocampus, memory, spatial navigation, all-optical interrogation, two-photon optogenetics, two-photon calcium imaging, virtual reality, inhibition, behavior

## Abstract

The hippocampus is crucial for spatial navigation and episodic memory formation. Hippocampal place cells exhibit spatially selective activity within an environment and have been proposed to form the neural basis of a cognitive map of space that supports these mnemonic functions. However, the direct influence of place cell activity on spatial navigation behavior has not yet been demonstrated. Using an ‘all-optical’ combination of simultaneous two-photon calcium imaging and two-photon optogenetics, we identified and selectively activated place cells that encoded behaviorally relevant locations in a virtual reality environment. Targeted stimulation of a small number of place cells was sufficient to bias the behavior of animals during a spatial memory task, providing causal evidence that hippocampal place cells actively support spatial navigation and memory.

## Highlights

• Two-photon optogenetics in VR enables targeted manipulation of place cell ensembles
• Activating specific place cell ensembles drives their spatially associated behavior
• Place cell stimulation inhibits endogenous place code expression and triggers remapping
• Direct evidence for a causal role of place cells in spatial navigation

## Graphical Abstract

**Figure:**
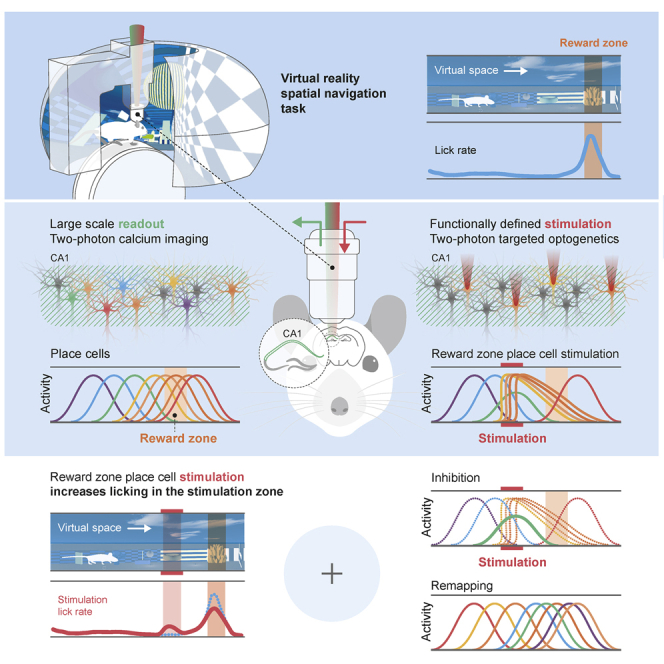

## Teaser

Selective stimulation of a small number of hippocampal place cells in mice provides causal evidence that hippocampal place cells actively support spatial navigation and memory.

## Introduction

The hippocampus is known to support both spatial navigation and episodic memory formation (Scoville and Milner, 1957; Morris et al., 1982; O’Keefe and Nadel, 1978; Ergorul and Eichenbaum, 2004). Many hippocampal pyramidal neurons exhibit location-specific firing and are referred to as place cells (O’Keefe and Dostrovsky, 1971). As a population, place cells are thought to form the basis of a cognitive map (Tolman, 1948; O’Keefe and Nadel, 1978; Eichenbaum et al., 1999; Schiller et al., 2015; Tavares et al., 2015), enabling memory-based navigation through mental and physical space. Place cell populations form largely unique maps to represent a given environment (Leutgeb et al., 2004; Alme et al., 2014) and remap, altering their firing properties in response to changes in that environment (Muller and Kubie, 1987; Bostock et al., 1991). Their reorganization to goal locations and subsequent reactivation predict spatial memory performance (Dupret et al., 2010; Xu et al., 2019) and the replay of place cell firing sequences (Skaggs and McNaughton, 1996; Lee and Wilson, 2002; Foster and Wilson, 2006; Diba and Buzsáki, 2007) has been linked to memory consolidation and retrieval (Girardeau et al., 2009; Jadhav et al., 2012; Fernández-Ruiz et al., 2019; Norman et al., 2019). Place cell activity often conjunctively encodes information about an experience, such as object identity (Wood et al., 1999; Moita et al., 2003; Komorowski et al., 2009; Robinson et al., 2017), time (Manns et al., 2007), valence (McKenzie et al., 2013, 2014), and retrospective or prospective location (Wood et al., 2000; Frank et al., 2000), supporting their likely role in episodic memory. Neurons with place field firing have been recorded from the human hippocampus (Ekstrom et al., 2003), and memory recall has been shown to correlate with their reactivation (Miller et al., 2013). Populations of hippocampal neurons also exhibit sequences of firing fields that tile the continuous dimensions of experiences involving modalities other than space (Eichenbaum et al., 1987; Pastalkova et al., 2008; MacDonald et al., 2011; Villette et al., 2015; Allen et al., 2016; Terada et al., 2017; Aronov et al., 2017; Omer et al., 2018; Danjo et al., 2018), and one prevailing view is that the generation of these sequences supports the formation of detailed episodic memories. The substantial body of place cell research is predominantly correlational; however, recent work has moved toward providing a direct link to their proposed function.

A group of elegant studies used immediate early gene-driven expression of an opsin to gain control over the activity of neurons that are highly active around a specific experience (Liu et al., 2012; Ramirez et al., 2013). They demonstrated that firing of hippocampal neurons that were active during contextual fear conditioning is both required and sufficient to later retrieve the associated behavior. However, these studies were not selective for neurons with specific coding properties and have been reported to predominantly involve neurons with wider context-specific coding rather than the spatial tuning that characterizes place cells (Tanaka et al., 2018). Previous work has shown that medial forebrain bundle (MFB) stimulation during sleep, triggered on place cell firing, increases the probability of the animal visiting the associated place field area during subsequent wakefulness (de Lavilléon et al., 2015). However, MFB stimulation is likely to reward the firing of not only place cells but also neurons in distributed brain systems that display correlated activity (Ji and Wilson, 2007; Tang et al., 2017). A causal role for place cell activity during memory-guided spatial navigation remains to be demonstrated.

Here, we utilize an “all-optical” combination of simultaneous two-photon calcium imaging and two-photon targeted optogenetics (Rickgauer et al., 2014; Packer et al., 2015; Carrillo-Reid et al., 2016; Mardinly et al., 2018; Marshel et al., 2019) in head-fixed mice performing a virtual reality spatial navigation task (Harvey et al., 2009; Schmidt-Hieber and Häusser, 2013; Rickgauer et al., 2014). This enabled us to record and stimulate populations of place cells with specific place field firing locations and assess their causal contribution to spatial behavior.

## Results

### All-Optical Manipulation of Place Cells during Virtual Spatial Navigation

**Figure 1:**
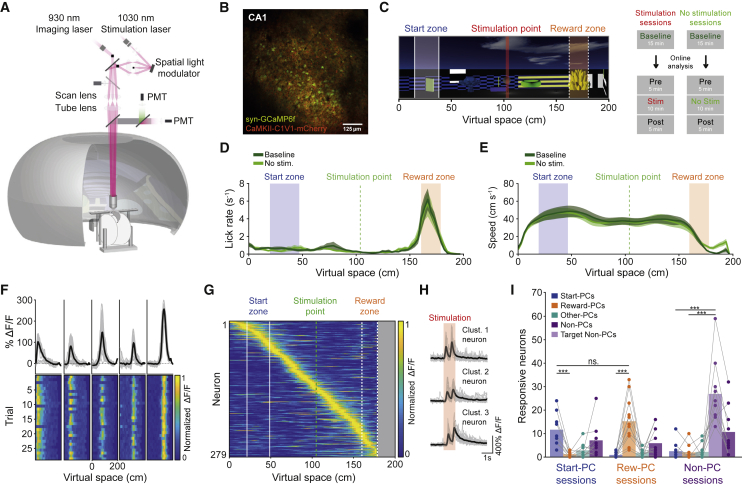
All-Optical Manipulation of Place Cells during Spatial Navigation in Virtual Reality (A) Schematic of head-fixed virtual reality setup and microscope design. (B) Example imaging field of view from CA1 *stratum pyramidale* showing neurons co-expressing GCaMP6f and C1V1. (C) Side-on view of the virtual reality linear track with start zone, reward zone, and stimulation point and schematic of the session structure. (D) Average lick-rate distribution across virtual space for the two behavioral epochs on no-stimulation days (n = 9 mice). (E) Average running speed distribution across virtual space for the two behavioral epochs on no-stimulation days. (F) Five simultaneously recorded place cells with ΔF/F traces across virtual space and ΔF/F heat plots across trials. (G) All place cells recorded from an example baseline epoch on a stimulation day and average ΔF/F across space for each neuron and ordered by peak location on the track. (H) Example photostimulation-targeted responsive neurons; black line shows photostimulus-triggered average response, and gray traces show individual trials; the two peaks correspond to the two 100 ms photostimulations of the cell. (I) Number of responsive neurons in Start-PC, Reward-PC, and Non-PC stimulation sessions where a single ensemble type was targeted for stimulation. ^∗^p < 0.05; ^∗∗∗^p < 0.0005; all error bars show SEM. See also Figures S1 and S2.

To simultaneously record and manipulate the activity of neurons in CA1 of the hippocampus, mice co-expressing GCaMP6f (Chen et al., 2013) and the excitatory opsin C1V1 (Yizhar et al., 2011) in CA1 pyramidal cells were implanted with an imaging window (Dombeck et al., 2010; Danielson et al., 2016). Animals were then head-fixed in a virtual reality environment (Figure 1A) in which we performed simultaneous two-photon imaging (Figure 1B) and two-photon targeted photostimulation of CA1 pyramidal cells. The head-fixed mice were trained to perform a virtual reality spatial navigation task, where they were required to wait 3 s and lick a minimum of three times in a specific rewarded zone on a virtual linear track to receive a sugar water reward (Figure 1C). Trials during which the animal licked more than 10 times outside the reward zone, or ran into the back wall of the 2-m track, resulted in failure and a white screen “timeout” penalty with a minimum duration of 10 s. A dark timeout with a minimum 5 s duration followed successful trials. In both cases, animals were required to stop licking for 3 s before a new trial began. Mice learned to perform this task (61.0% ± 18.8% correct during baseline behavior, n = 21 sessions), stopping and licking specifically in the rewarded zone (Figures 1D and 1E; p < 10^−9^ in versus out of reward-zone lick rate, p < 10^−7^ in versus out of reward-zone running speed, paired t test, n = 21 sessions).

We performed two-photon calcium imaging during the behavior, recording the GCaMP6f signal from the somata of a large population of CA1 neurons (605 ± 177 neurons per session) and then using this to identify those with a place field on the virtual track (Figures 1F and 1G; Dombeck et al., 2010). Many recorded neurons had place fields (141 ± 77 neurons, 27.4% ± 10.6% of neurons, similar to Dombeck et al., 2010), and these field locations were distributed over the entirety of the track (Figures 1G, S5B, and S5C). We then assessed the role of place cell activity in guiding spatial navigation using targeted two-photon optogenetic activation of specific place cells (Rickgauer et al., 2014). We hypothesized that driving activity in a population of similarly tuned place cells would bias mouse behavior toward that which is normally exhibited in the location of those cells’ place fields. Such a result would provide evidence for a causal role for place cell activity in guiding spatial behavior and supporting spatial memory. We categorized place cells with fields that covered >50% of the reward zone of the virtual track as reward-zone place cells (Reward-PCs) and those covering an area near the beginning of the track as start zone place cells (Start-PCs). The start zone was slightly larger than the reward zone to enable an equivalent population of neurons to be categorized and targeted for each group (44.4 ± 15.7 Start-PCs and 48.8 ± 26.1 Reward-PCs per session). Reward-PC activity was spatially associated with a high lick rate and decelerating running speed, while Start-PCs were active during periods of low lick rate and stable high running speed (Figures 1D, 1E, and 1G). During each session, animals initially ran trials over a 15-min baseline epoch, and the data were then analyzed to identify and target neurons for stimulation. This was followed by a further 5 min of no-stimulation trials (Pre) and 10 min of stimulation trials, where one neural ensemble each session (Start-PCs, Reward-PCs, or Non-place cells) was activated as the animal crossed the central stimulation point on each trial, followed by 5 min with no stimulation (Post). During control sessions replicating the temporal structure of the experiment but lacking stimulation, task performance, lick rate, and running speed remained stable over two baseline epochs separated by an hour delay (Figures 1D and 1E; delta percentage of correct trials p = 0.55, two-sided rank-sum test, lick rate p = 0.91, running speed p = 0.73, Wilcoxon signed-rank test, n = 12 sessions from 9 mice). There was no detectable difference in baseline behavior between sessions where the different place cell groups were stimulated (delta percentage of correct trials p = 0.41, two-sided rank-sum test, lick rate p = 0.30, running speed p = 0.81, Wilcoxon signed-rank test, n = 9 Start-PC sessions, 7 mice and 12 Reward-PC sessions, 8 mice).

**Figure S1:**
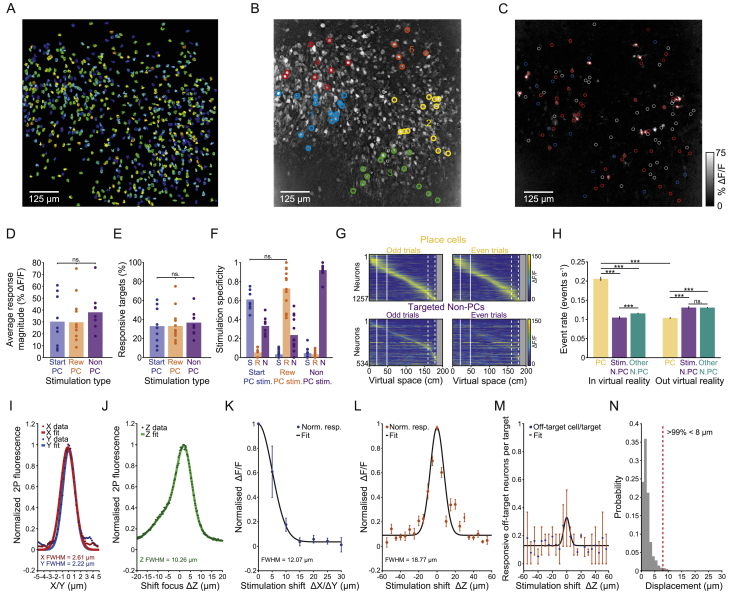
Two-Photon Optogenetic Stimulation of Targeted Populations, Non-PC Control Group Characterization, Stimulation Resolution, and Brain Motion Quantification, Related to Figure 1 (**A**) Regions of interest extracted from one example session FOV. (**B**) 5 spatially clustered groups of Reward-PCs overlaid on a correlation image of the GCaMP6f signal from the imaging FOV, pixels values are weighted by their signal correlation with neighboring pixels. (**C**) Stimulation triggered average ΔF/F average intensity image from artifact subtracted recording frames during stimulation, taken from the same session as (**A**), red circles indicate targeted Reward-PCs, white circles denote non-targeted Start-PCs and blue circles denote stimulation zone place cells. (**D**) Average response magnitude across the population of responsive target neurons from each session. (**E**) Percentage of targets deemed responsive from each session. (**F**) Stimulation specificity for each experimental session, defined as the number of responsive neurons in the group divided by the sum of the number of responsive neurons across all groups. (**G**) Place cell population average ΔF/F across virtual space for targeted non-place cells and place cells from the same sessions, neurons in both plots are ordered by their peak ΔF/F in odd trials. (**H**) Event rates of place cells, targeted non-place cells and other non-place cells both in the virtual reality world and during the inter-trial interval, events were defined as the ΔF/F reaching 3 SD above the mean, error bars are SEM. (**I**) X and Y axis optical point spread function measurement for a stimulation beamlet. (**J**) Axial optical point spread function measurement for a stimulation beamlet. (**K**) All-optical physiological resolution along the X/Y axis as measured by the normalized GCaMP6f signal resulting from stimulation at different X/Y displacements from the target neurons, n = 21 neurons from 3 mice, error bars are 95% confidence interval. (**L**) Axial all-optical physiological resolution as measured by the normalized GCaMP6f signal resulting from stimulation at different Z displacements from the target neurons, n = 23 neurons from 2 mice, error bars are 95% confidence interval. (**M**) The fraction of off-target neurons per targeted cell when stimulating at different axial displacements, n = 2 sessions from 2 mice. (**N**) X/Y displacement resulting from brain motion during all stimulation epochs, determined by the translation required to maximize the correlation between each frame and an averaged image of the FOV.

To increase the number of place cells we could optogenetically activate, we used a large imaging field of view and split the selected neurons into five spatial clusters, which were rapidly activated in a sequence (Figures S1A and S1B). During the stimulation epoch, we triggered optogenetic stimulation of these clusters when the mouse crossed the stimulation point of the virtual track (Figure 1C). The clusters were activated sequentially for 100 ms each, cycling through twice for a total stimulation duration of 1 s. Two-photon optogenetic stimulation triggered robust and specific activation of many of the targeted neurons (Figures 1H and 1I; Figures S1C–S1F). Responsive neurons were defined as those exhibiting robust GCaMP6f signals (>40% ΔF/F) following stimulation on at least 30% of trials. There were far more responsive neurons in our targeted place cell population than the non-targeted place cell category (Figure 1I, 13.71 ± 8.53 versus 1.05 ± 1.16, p = 3.9 × 10^−3^ for Start-PC sessions and p = 4.89 × 10^−4^ for Reward-PC sessions, Wilcoxon signed-rank test). There was no detectable difference in the number of responsive place cells in Start-PC and Reward-PC sessions (p = 0.41, two-sided rank-sum test). In order to further rule out the possibility that general increased network excitation led to any behavioral alterations, a larger number of neurons were stimulated and responded during non-place cell (Non-PC) stimulation control sessions (Figures 1I, S1G, and S1H, p = 0.0013 and p = 0.0087 for Start-PC and Reward-PC sessions, Kruskal-Wallis with Dunn’s test, n = 10 sessions, 6 mice).

**Figure S2:**
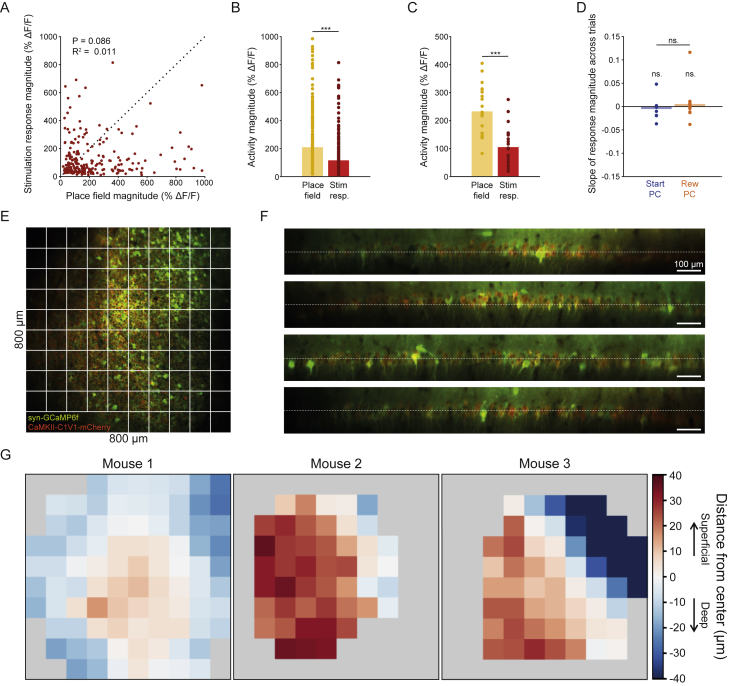
Stimulated Place Cell Activation Was within Physiological Range, Stable across Trials, and Consisted of a Mixture of Deep/Intermediate/Superficial CA1 Pyramidal Neurons, Related to Figure 1 (**A**) Place field magnitude against response magnitude for all responsive targeted place cells. (**B**) Average place field peak magnitude and response window magnitude for all responsive targeted place cells. On average our neurons’ natural place fields exhibited a larger response than that driven by our stimulation, p = 5.88 × 10^−16^, two-sided rank-sum test. (**C**) Average place field peak magnitude and stimulation response magnitude for all responsive place cells averaged within each session. Our population average natural place field magnitude was larger than that driven by our stimulation, p = 3.31 × 10^−5^, two-sided rank-sum test, n = 21 sessions. (**D**) The slope of a linear fit to the response magnitude of the population of responsive neurons across trials from each session. These slopes did not differ from zero in either place cell stimulation group, and there was no difference in slope between Start-PC and Reward-PC sessions. (**E**) Example imaging FOV taken from one mouse. (**F**) Four example slices through the z stack taken from the same area of that mouse, dashed white line indicates the selected imaging plane. (**G**) Color maps depicting the deep/superficial location of neurons across the imaging FOV taken from 3 separate mice, gray indicates no neurons were present in that area.

We quantified the number of off-target responsive neurons across the field of view in every experiment, as well as the optical and physiological resolution of our system (Figures S1I–S1L), which indicated that some additional off-target neurons are likely to have been stimulated outside the imaging plane. We also estimated the number of responsive off-target cells when the stimulation beamlets were at different axial displacements from the imaging field of view (Figure S1M) and characterized the amount of brain motion during stimulation sessions (Figure S1N). There were no detectable differences in the magnitude of response or the proportion of responsive targets during our different stimulation session types (Figures S1D and S1E; p = 0.33 and p = 0.59, respectively; Kruskal-Wallis with Dunn’s test) and in the stimulation specificity of our place cell groups (Figure S1F, p = 0.18, two-sided rank-sum test). The stimulation efficacy remained stable across trials during place cell stimulation sessions (Figure S2D). On average our stimulation evoked less activity in individual neurons than we observed endogenously during place field traversals, suggesting that our manipulation remained within physiologically realistic ranges (Figures S2A–S2C). The large field of view used in our experiments meant that we recorded and manipulated the activity of neurons across the depth of the curved CA1 cell layer (Figures S2E–S2G), interrogating both deep and superficial CA1 pyramidal cells (Mizuseki et al., 2009b; Valero et al., 2015; Danielson et al., 2016; Mallory and Giocomo, 2018).

### Reward-Zone Place Cell Activation Drives Spatially Associated Behavior

**Figure 2:**
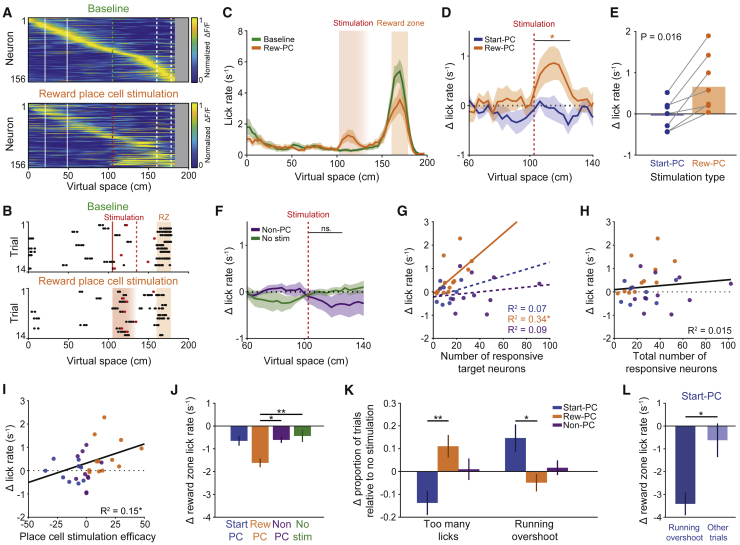
Targeted Stimulation of Reward-Zone Place Cells Drives Reward-Zone-Related Behavior (A) Place cell population average ΔF/F across virtual space from an example Reward-PC stimulation session; top is the baseline epoch and bottom is the stimulation epoch, and neurons in both plots are ordered and normalized by their peak during the baseline epoch. (B) Raster plot of licking across space from the baseline and stimulation epochs of one example reward place cell stimulation session. Red circles denote licks that caused a trial to end in failure by crossing the threshold for the number of licks allowed outside of the reward zone. Only trials where the animal reached the stimulation point are shown, and trial numbers have been matched between epochs by taking trials from the end of the baseline epoch. (C) Average lick-rate distribution across space for baseline and Reward-PC stimulation epochs across all Reward-PC stimulation sessions. Note the increased lick rate during stimulation. (D) Change in lick rate from baseline across space during Reward-PC and Start-PC stimulation epochs, averaged within and then across animals (n = 7 mice). (E) Summary of the within mouse change in licking caused by Reward-PC and Start-PC stimulation. (F) Change in lick rate from baseline during non-place cell stimulation sessions (n = 6 mice) and no-stimulation control sessions (n = 9 mice). (G) Correlation between number of responsive target population neurons and change in lick rate from baseline for Reward-PC, Start-PC, and Non-PC sessions. (H) The total number of stimulated neurons across all sessions does not correlate to the change in licking. (I) Place cell stimulation efficacy correlates with the observed change in licking (see STAR Methods). (J) Change in reward-zone lick rate during stimulation epoch or no-stimulation equivalent. (K) Summary of trial outcome changes during stimulation experiments relative to baseline data. (L) Change in reward-zone lick rate separately for running overshoot and other trials during Start-PC stimulation sessions where there was an increase in running overshoots (n = 6 sessions). ^∗^p < 0.05, ^∗∗^p < 0.01; all error bars show SEM.

We first examined the effects of specific place cell stimulation on the lick distribution across space as the mouse traversed the environment. Strikingly, we found that driving Reward-PC activity prior to the reward zone and earlier in the virtual track than their endogenous firing fields (Figure 2A), resulted in a coincident increase in lick rate relative to the behavioral baseline (Figures 2B and 2C; p = 0.0098, Wilcoxon signed-rank test, n = 12 sessions, 8 mice), biasing the animal’s behavior toward that which was normally exhibited in the reward zone. The change in lick rate due to Reward-PC stimulation was greater than any change during Start-PC stimulation sessions (Figures 2D and 2E; p = 0.016, Wilcoxon signed-rank test, 7 mice). Furthermore, we found no increase in lick rate during Start-PC stimulation, Non-PC stimulation, or no-stimulation control sessions (Figure 2F; p = 1, p = 0.49, and p = 0.84, n = 9, 10, and 12 sessions, respectively; Wilcoxon signed-rank test). The magnitude of the increase in lick rate caused by Reward-PC stimulation correlated with the number of stimulation-responsive target neurons (Figure 2G, R^2^ = 0.34, p = 0.046, n = 12 sessions, 8 mice). There was no relationship when targeting Start-PCs or Non-PCs, and the total number of neurons that were stimulated in a session was not predictive of the change in stimulation zone licking rate (Figure 2H, R^2^ = 0.015, p = 0.51, 31 sessions). Additionally, we found that place cell stimulation efficacy, a metric combining the stimulation specificity to one place cell population, the number of neurons responding, and the magnitude of the response, was related to altered licking (Figure 2I, R^2^ = 0.15, p = 0.032). We observed a decrease in on-target reward-zone licking following Reward-PC stimulation when compared to our controls (Figure 2J, p = 0.0015 and p = 0.013 against no-stimulation and Non-PC sessions, Kruskal-Wallis with Dunn’s test). Together, these results demonstrate a specific and causal role for place cell activity in the retrieval of a learned spatial behavior.

### Effects of Place Cell Stimulation on Trial Outcome and Running Behavior

**Figure 3:**
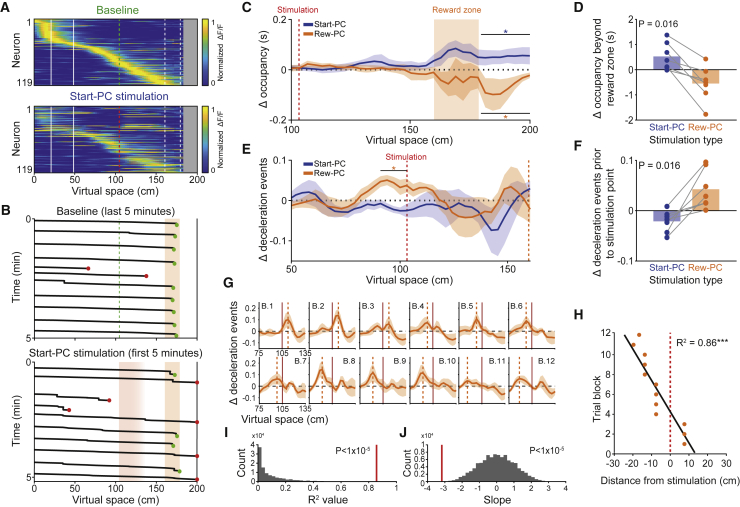
Influence of Place Cell Stimulation on Running Behavior (A) Place cell population average ΔF/F across virtual space from an example Start-PC stimulation session, top is baseline epoch and bottom is stimulation epoch, and neurons in both plots are ordered and normalized by baseline peak. (B) Spatial trajectory data from the last 5 min of an example baseline epoch and first 5 min of the subsequent Start-PC stimulation epoch; green circles denote correct trials, red circles denote incorrect trials, and orange area depicts the reward zone. (C) Change in spatial occupancy from baseline across space during Reward-PC and Start-PC stimulation epochs, averaged within and then across animals (n = 7 mice). (D) Summary of the within mouse change in occupancy beyond the reward zone caused by Reward-PC and Start-PC stimulation (n = 7 mice). (E) Change in the number of deceleration events from baseline across space during Reward-PC and Start-PC stimulation epochs. (F) Summary of the within mouse change in deceleration events before the stimulation point caused by Reward-PC and Start-PC stimulation (n = 7 mice). (G) Change in the number of deceleration events from baseline during Reward-PC stimulation epochs relative to the stimulation point, 3 trial blocks in each panel with a sliding window approach, averaged within and then across animals (n = 7 mice); dashed orange lines mark the peak of the increase in deceleration events, solid red lines marks the stimulation point. (H) Peak location of the increase in deceleration events across trial blocks (Pearson’s correlation). (I) Chance distribution of correlation R^2^ values generated from 100,000 shuffles of trial block order and observed value from the data in (H) (red line). (J) Same as in (I) but for the slope of a linear fit. ^∗^p < 0.05, ^∗∗∗^p < 0.001; all error bars show SEM. See also Figure S3.

**Figure S3:**
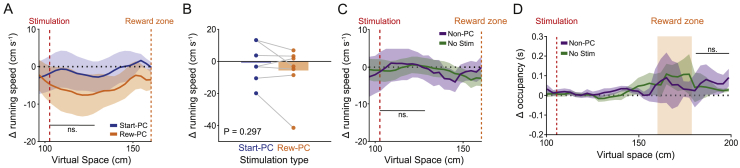
Effect of Stimulation on Running Speed, Related to Figure 3 (**A**) Animal-wise delta running speed at the stimulation point between baseline and stimulation epochs for Start-PC and Reward-PC stimulation sessions. (**B**) Summary of the within animal delta running speed resulting from stimulation. (**C**) Delta running speed at the stimulation point between baseline and stimulation epochs for Non-PC stimulation sessions and no stimulation control sessions. (**D**) Change in spatial occupancy from baseline during Non-PC stimulation and no stimulation control epochs. All error bars show SEM.

We next examined stimulation-induced changes in trial outcome relative to baseline levels. The increased lick rate that resulted from Reward-PC stimulation was accompanied by an increase in the proportion of failed trials where the mouse licked too many times outside the reward zone, when comparing Reward-PC and Start-PC stimulation sessions (Figure 2K, p = 0.008, two-sided rank-sum test). Interestingly, stimulating Start-PCs in the middle of the track (Figure 3A), later than their endogenous firing fields, caused an increase in the proportion of trials where the animal ran beyond the reward zone, relative to Reward-PC stimulation sessions (Figures 2K and 3B, p = 0.018, two-sided rank-sum test). During Start-PC sessions where the number of running overshoot trials increased, there was a marked decrease in licking in the reward zone during running overshoot trials (Figure 2L, p = 0.01, two-sided rank-sum test, n = 6 sessions, 4 mice), consistent with the animals’ failure to correctly identify the location. In agreement with the increased number of running overshoot trials, Start-PC stimulation also caused an increase in the average occupancy in the area beyond the reward zone (Figure 3C, p = 0.031, Wilcoxon signed-rank test, n = 7 mice), whereas Reward-PC stimulation reduced this occupancy (Figure 3C, p = 0.016, Wilcoxon signed-rank test, n = 7 mice), with the delta occupancy clearly differing between Start-PC and Reward-PC sessions (Figure 3D, p = 0.016, two-sided Wilcoxon signed-rank test, n = 7 mice). There was no significant change in occupancy beyond the reward zone during Non-PC or no-stimulation control sessions (Figure S3D, p = 0.38 and p = 0.25, Wilcoxon signed-rank test, n = 10 sessions, 6 mice and 12 sessions, 10 mice). Additionally, Start-PC stimulation caused an increase in reward-zone occupancy (Figure 3C, p = 0.031, Wilcoxon signed-rank test, n = 7 mice), driven by trials in which the animal stopped within the reward zone but failed to lick to trigger timely reward delivery (Figure 3B) and a reduction in the proportion of trials where the animal failed due to excessive licking prior to the reward zone (Figure 2K). There was no significant change in occupancy in the reward zone during Reward-PC, Non-PC, or no-stimulation control sessions (Figures 3C and S3D, p = 0.31, p = 0.85, and p = 0.06, Wilcoxon signed-rank test). Collectively, these findings demonstrate a behavioral effect of Start-PC stimulation that manifests after the stimulation has ceased and suggests a lasting impact on neural activity that is not fully reset by the visual cues in the environment.

We further investigated the effect of stimulation on the running behavior of our animals and detected no significant changes in average running speed over the stimulation zone (Figures S3A–S3C). Curiously, we observed an increased number of deceleration events prior to the stimulation point specifically during Reward-PC stimulation sessions (Figures 3E and 3F, n = 12 sessions, 8 mice; events defined as when the animal’s acceleration was 2 standard deviations below the mean value; p = 0.0078 for Reward-PC, p = 0.078, p = 0.81, and p = 0.19 for Start-PC, Non-PC, and No stimulation, respectively; Wilcoxon signed-rank test). We found that this increase occurred during the stimulation in early trials and progressively moved to earlier locations on the track, such that in later trials the animals decelerated prior to the onset of stimulation (Figures 3G–3J). This alteration in running behavior suggests that Reward-PC stimulation triggers changes in the network that cause the animal to anticipate the stimulation zone.

### Place Cell Stimulation Interacts with the Endogenous Code

**Figure 4:**
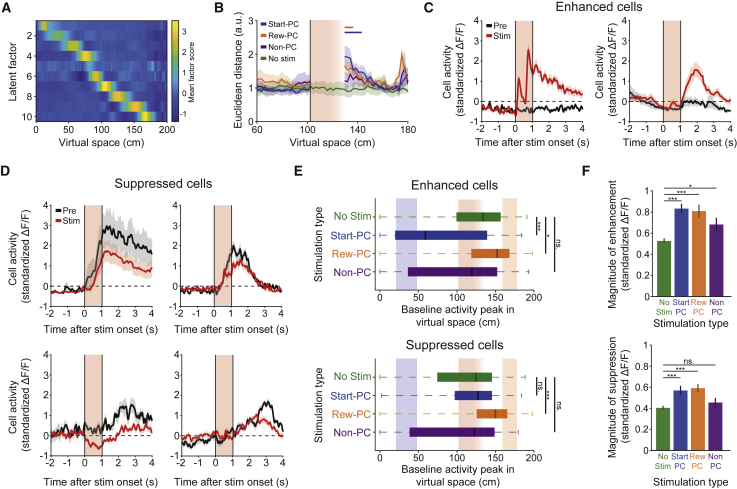
Targeted Stimulation of Place Cells Interacts with Endogenous Activity (A) Factor analysis yields a low-dimensional representation of population dynamics. Shown is a representative example of trial-averaged baseline activity. The coordinated activity of groups of correlated place cells is reflected in the spatial tuning of latent factors. (B) Euclidean distance between mean latent trajectories for stimulation trials and the immediately preceding pre-epoch trials; lines along the top show consecutive bins that are significantly different after stimulation when compared to before (p < 0.05, two-sided rank-sum test). The divergence of trajectories comprising the second peak at the end of the reward zone was not statistically significant. Photostimulation occurred during the red shaded area (the data during this period were not included in the analysis). (C) Standardized stimulus triggered average ΔF/F traces from two example neurons that were identified as being enhanced during stimulation trials; the red area depicts the stimulation duration. (D) Standardized stimulus triggered average ΔF/F traces from four example neurons that were identified as being suppressed during stimulation trials. (E) The baseline spatial tuning of neurons that were identified as either enhanced or suppressed during stimulation or the no-stimulation equivalent; plots show median and interquartile range. (F) The magnitude of enhancement or suppression was greater during place cell stimulation than during the equivalent epochs from no-stimulation control sessions. The magnitude of suppression following Non-PC stimulation was similar to no-stimulation control data. ^∗^p < 0.05, ^∗∗∗^p < 0.001; all error bars show SEM. See also Figure S4.

The behavioral effects of activating place cells were generated by stimulating remarkably few neurons (15.3 ± 9.5 responding Reward-PC neurons on average). As previous work has shown that the stimulation of a single place cell can affect the activity of other place cells (Rickgauer et al., 2014), we hypothesized that the behavioral effect of our activation could be facilitated by interactions with the endogenous place cell code. To investigate this, we examined the trajectories of place cell populations in a low-dimensional latent space using factor analysis (see STAR Methods and Rickgauer et al., 2014). By definition, these populations included targeted cells for Start-PC and Reward-PC stimulation sessions but not for Non-PC stimulation sessions. Consistent across experimental sessions, we found that latent variables were spatially tuned, reflecting the coordinated modulation of place cell aggregates along the virtual track (Figure 4A). To assess the effect of stimulation on network dynamics, we compared the mean latent trajectories from stimulation trials to those of the immediately preceding block of control trials. All imaging frames acquired during stimulation were excluded from this analysis to avoid contamination from possible photo-artifacts. In spatial positions immediately following the excluded stimulation zone, we observed a pronounced divergence of trajectories for Start-PC and Reward-PC stimulation conditions but no detectable differences in Non-PC or no-stimulation conditions (Figure 4B). This divergence of latent trajectories observed could be driven by activation of the targeted cells alone, or in conjunction with disruption of the competing endogenous spatial representation. Repeating the analysis with exclusion of directly targeted and neighboring cells (within a 30-μm radius of a targeted cell body), we could no longer detect significant deviations in the trajectories following the stimulation zone. However, due to the number and spatial distribution of our stimulation targets, these exclusions involved removing a large proportion of the population, including almost all cells encoding for the start or reward locations and thus do not definitively rule out contributions from non-targeted cells to the network perturbation. To further explore this possibility, we quantified the responses of individual place cells with high factor loadings (both targeted and non-targeted) in the spatial bins following the stimulation zone, excluding those already showing between-epoch differences in activity prior to the stimulation zone. We thus identified subpopulations of neurons from each condition exhibiting strongly suppressed or enhanced spatial responses (defined as the first and last deciles of the distribution of activity differences within sessions), which corresponded to long-lasting enhanced (Figure 4C) or suppressed (Figure 4D) activity when aligned to stimulus onset.

For Start-PC and Reward-PC stimulation conditions, enhanced cells were predominantly tuned to the location of the targeted populations’ place fields (Figure 4E), confirming the specificity of our stimulation. As expected, the magnitude of enhancement was larger in stimulated conditions than in the no-stimulation control (Figure 4F, p = 2.45 × 10^−11^, p = 6.07 × 10^−6^, and p = 0.02 for Start-PC, Reward-PC, and Non-PC, respectively, two-sided rank-sum test). Detection of activity suppression in our analysis requires cells to be endogenously active near the stimulation zone. This detection bias is reflected in the localized suppressed-cell tuning distributions of Start-PC, Non-PC, and no-stimulation conditions (Figure 4E). For Reward-PC stimulation, however, the suppressed-cell tuning distribution was shifted toward the reward zone. This is due in part to the presence of a considerable fraction of directly targeted reward-zone cells (23/87 suppressed cells). These cells were typically associated with a low-magnitude response to direct stimulation, followed by a reduction of in-field activity that otherwise begins to increase after the stimulation zone (Figure 4D, bottom-right panel), consistent with previous observations (Rickgauer et al., 2014). Although the tuning of suppressed cells was otherwise broadly similar across conditions, the magnitude of suppression was greater after Start-PC or Reward-PC stimulation, compared to the no-stimulation control (Figure 4F, p = 2.6 × 10^−3^ and p = 1 × 10^−4^ for Start-PC and Reward-PC, respectively, two-sided rank-sum test). We did not observe this increased suppression during Non-PC stimulation sessions (p = 0.82, two-sided rank-sum test), even though considerably more neurons were activated.

**Figure S4:**
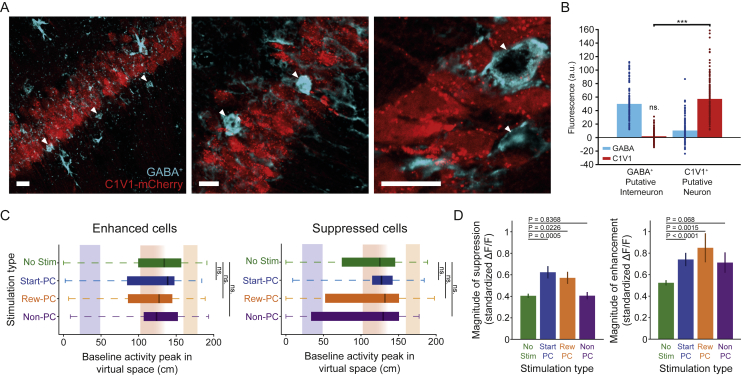
Opsin Expression Was Largely Specific to Excitatory Neurons and Activity Suppression Is Still Evident When Discounting Stimulation Targeted Neurons and Those within Range of Off-Target Activation, Related to Figure 4 (**A**) Immunohistochemistry identified GABA^+^ putative interneurons (white arrowheads), which did not exhibit opsin-associated mCherry signal, scale bars show 15 μm. Note that some GABA^+^ ROIs will correspond to astrocytes, and these have been excluded from the quantification based on their morphology. (**B**) Quantification of background subtracted fluorescence signal within GABA^+^ and C1V1^+^ ROIs. The GABA^+^ ROIs had background subtracted opsin-associated signal that did not differ from zero (p = 0.1, signed-rank test n = 114) and which was significantly lower than that in C1V1^+^ ROIs (p = 6.15 × 10^−20^, signed-rank test, n = 114 GABA^+^ and 157 C1V1^+^ ROIs). (**C**) The spatial tuning of neurons which were identified as either enhanced or suppressed with targets and proximal neurons excluded, plots show median and interquartile range. (**D**) The magnitude of enhancement or suppression in identified neurons during place cell stimulation and the equivalent epochs during no stimulation control sessions. The increase in suppression observed during place cell stimulation remained when targeted and proximal neurons were excluded from the analysis. Error bars show SEM.

We confirmed through immunohistochemistry that opsin expression was predominantly restricted to pyramidal neurons, such that the photostimulation of interneurons was highly unlikely (Figures S4A and S4B). Combined with the relative lack of suppression observed following Non-PC stimulation, where more cells were targeted and thus more light applied to the tissue, this indicates that the increased suppression observed following place cell stimulation was not driven by the direct activation of interneurons. These observations of place cell suppression, which are robust to removal of all targeted and neighboring cells from the comparison to control (Figures S4C and S4D), imply a polysynaptic propagation of place cell stimulation throughout the place cell network. Our results are consistent with a model in which stimulation creates a bias in representation toward the targeted location, and the downstream effects of this are facilitated by inhibition of the endogenous spatial code. This could be mediated by the recruitment of recurrent local inhibitory neurons (Freund and Buzsáki, 1996; Pouille and Scanziani, 2004; Grienberger et al., 2017; McKenzie et al., 2019), which cause disynaptic inhibition of the endogenous place cell population. The observed increase in suppression following place cell stimulation, but not Non-PC stimulation, suggests that place cells may preferentially interact with members of the same map, communicating through local interneurons to guide network dynamics.

### Lasting Network and Behavioral Impact of Place Cell Activation

**Figure 5:**
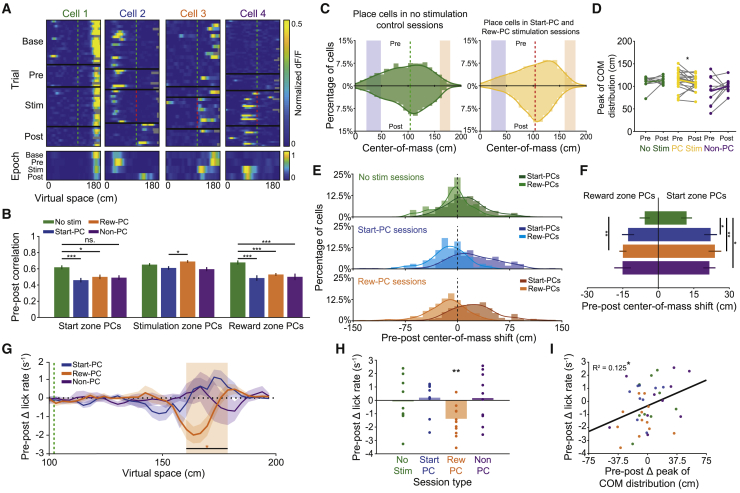
Stimulation-Driven Remapping Influences Spatial Behavior (A) Trial-wise normalized ΔF/F heat plots for 4 neurons from baseline, Start-PC, Reward-PC, and Non-PC stimulation sessions; the track is cropped at 180 cm due to low occupancy data beyond the reward zone. Only trials where the mouse traversed at least 150 cm of the track are shown. (B) Single-cell correlation values for average pre-post epoch place maps across sessions and split by baseline place field location. (C) Distributions of place field center-of-mass for pre- and post-epochs during no-stimulation and place cell stimulation sessions. Note the shift toward the center of the track after stimulation of the place cell network. (D) Pre-post place field center-of-mass distribution peak differences across session type. (E) Center of mass shifts for all Start-PCs and Reward-PCs during no-stimulation, Start-PC stimulation, and Reward-PC stimulation sessions. (F) Average single-cell center-of-mass shifts for Start-PCs and Reward-PCs across session types. (G) Pre-post change in lick distribution across space averaged across sessions. (H) Summary of the change in lick rate within the reward area between pre- and post-epochs for different session types, licking was decreased following Reward-PC stimulation. (I) Correlation between the change in reward-zone lick rate and the shift in place cell distribution peak across all session types. ^∗^p < 0.05, ^∗∗^p < 0.01, ^∗∗∗^p < 0.001; all error bars show SEM. See also Figure S5.

**Figure S5:**
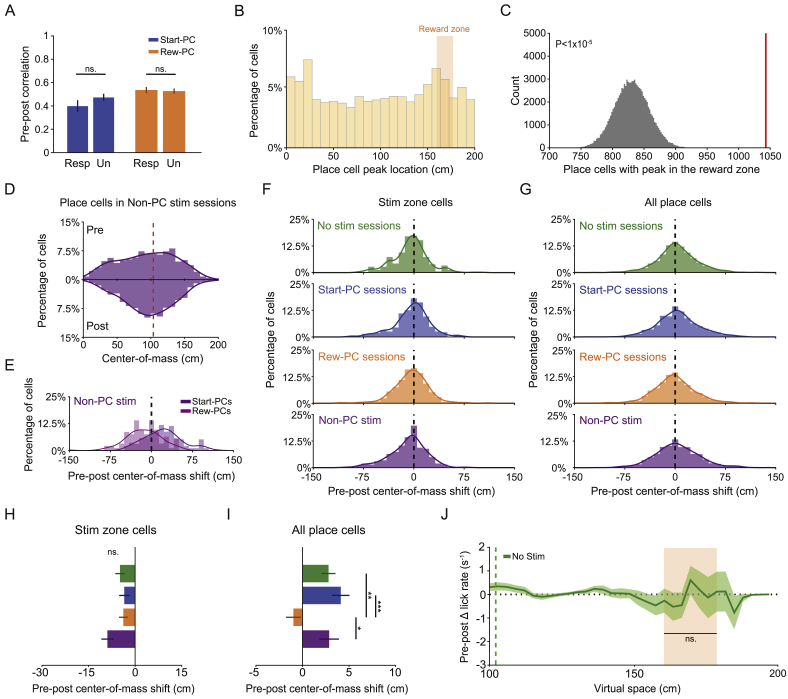
Stimulation-Driven Remapping Controls, Related to Figure 5 (**A**) Pre-post epoch spatial activity profile correlation for responsive and unresponsive Start-PCs and Reward-PCs, demonstrating that we did not observe more remapping in responsive neurons (p = 0.14 Start-PC and p = 0.69 Reward-PC, two-sided rank-sum test). (**B**) Calcium trace peak ΔF/F value location across virtual space for place cells identified during the baseline epoch of stimulation days. (**C**) Shuffled chance distribution of the number of place cells with peaks inside the reward zone and real data value (red line). Shuffle distribution generated by randomly translating each place cells average ΔF/F trace within the spatial range of the track. (**D**) Place cell center of mass distributions for all place cells in pre and post epochs of Non-PC stimulation days. (**E**) Pre-post center of mass shift for Start-PCs and Reward-PCs during Non-PC stimulation sessions. (**F**) Pre-post center of mass shift for stimulation zone place cells across all session types. (**G**) Pre-post center of mass shift for all place cells across different session types. (**H**) Summary of the stimulation zone place cell pre-post center of mass shifts, no differences were found (Kruskal-Wallis test). (**I**) Summary of the pre-post center of mass shift for all neurons, the shift was different when comparing Reward-PC sessions to all other session types, p = 0.0013, p = 0.0002 and p = 0.0145 for No stim, Start-PC and Non-PC respectively, Kruskal-Wallis with Dunn’s test. (**J**) Delta lick rate between pre and post equivalent epochs for non-stimulation days. All error bars show SEM.

Finally, we investigated whether our targeted stimulation had any long-lasting effects on animal behavior, or the place cell network. Interestingly, there was a decrease in the on-target lick rate in the reward zone specifically during post-stimulation epochs that followed Reward-PC stimulation (Figures 5G, 5H, and S5J; p = 0.0049 for Reward-PC, p = 0.57, p = 0.77, and p = 1 for Start-PC, Non-PC, and No stimulation, respectively; Wilcoxon signed-rank test of delta pre-post lick distribution, with Bonferroni correction). We did not observe any persistent changes in the stimulation zone lick rate (p = 0.55, p = 0.22, and p = 0.31, Wilcoxon signed-rank test on delta distribution for Reward-PC, Start-PC, and Non-PC, respectively), running speed (p = 0.25, p = 1, and p = 0.69, Wilcoxon signed-rank test), occupancy beyond the reward zone (p = 0.31, p = 0.22, and p = 0.85, Wilcoxon signed-rank test), or occurrence of deceleration events prior to the stimulation point (p = 0.84, p = 0.47, and p = 0.69, Wilcoxon signed-rank test).

To assess stimulation-related changes in local network activity, we compared the spatial tuning of place cells in the pre- and post-stimulation epochs. At the single-cell level, neurons remapped, changing their spatial tuning in a variety of ways (Figure 5A). This remapping was reflected in a decrease in the single-cell pre-post place map correlation for stimulation sessions, when compared to the equivalent no-stimulation control session values (Figure 5B, n = 2,536, 1,665, 1,962, and 1,199 place cells for no-stimulation, Start-PC, Reward-PC, and Non-PC sessions, respectively). Remapping was present in both Start-PC (p = 5.6 × 10^−4^, p = 0.022, and p = 0.055 for Start-PC, Reward-PC, and Non-PC stimulation, respectively; Kruskal-Wallis with Dunn’s test) and Reward-PC groups (p = 4.9 × 10^−6^, p = 5.1 × 10^−6^, and p = 5.1 × 10^−4^ for Start-PC, Reward-PC, and Non-PC stimulation, respectively; Kruskal-Wallis with Dunn’s test) during both place cell stimulation session types and was not greater in stimulation responsive neurons (Figure S5A, p = 0.27 and p = 0.69 for Start-PC and Reward-PC groups, respectively, Wilcoxon rank-sum test), demonstrating a lack of specificity. Place cells with fields overlapping the stimulation area did not undergo sufficient remapping to produce a decrease in pre-post place map correlation (Figures 5B, S5F, and S5H, p = 0.71, p = 0.55, and p = 0.63 for Start-PC, Reward-PC, and Non-PC stimulation, respectively; Kruskal-Wallis with Dunn’s test), suggesting that one characteristic of this remapping was a tendency to shift firing toward the stimulated area of the track during the post-stimulation epoch. Indeed, when calculating the distribution of place fields along the track before and after place cell stimulation epochs, the overrepresentation of the rewarded area that was present prior to place cell stimulation was altered to a distribution that peaked in the center of the track (Figures 5C, 5D, S5D, and S5E, p = 0.65, p = 0.030, and p = 0.19 for no stimulation, PC, and Non-PC stimulation, respectively; Wilcoxon signed-rank test on peak location of center-of-mass distribution). Start-PCs underwent a greater pre-post field shift than in no-stimulation control sessions following every stimulation type (Figures 5E and 5F; p = 0.019, p = 0.0080, and p = 0.042 for Start-PC, Reward-PC, and Non-PC stimulation, respectively; Kruskal-Wallis with Dunn’s test). This shift only reached significance for Reward-PCs during Reward-PC stimulation sessions, although there was a similar average magnitude shift following other stimulation types (p = 0.23, p = 0.0038, and p = 0.085 for Start-PC, Reward-PC, and Non-PC stimulation, respectively; Kruskal-Wallis with Dunn’s test). When relating remapping to any change in on-target lick rate, we found that the shift of the place cell distribution peak correlated with changes in on-target licking, such that the greater the shift away from the rewarded zone the greater the decrease in lick rate (Figure 5I, R^2^ = 0.12, p = 0.026; Pearson’s correlation, n = 41 sessions). This result supports previous work suggesting that the migration of place fields to over-represent reward locations supports spatial memory (Dupret et al., 2010). Taken together, these results indicate that targeted optogenetic stimulation of specific place cells triggered remapping of the hippocampal representation of space. This remapping was not limited to targeted neurons, and the resulting change in place field distribution was linked to a decrease in goal-directed licking.

## Discussion

We have used an “all-optical” strategy, combining simultaneous two-photon calcium imaging and two-photon holographic optogenetics, to functionally define a population of place cells in the hippocampus and selectively drive their activity, while observing the effects on navigational behavior and local circuit activity. Remarkably, targeted optogenetic stimulation of specific place cell populations was sufficient to bias behavior toward that associated with the location of their place fields. In addition, we characterized changes in network activity driven by our stimulation, as well as the resulting place field remapping and its relationship to changes in goal-directed licking. These findings demonstrate a causal role for place cell activity in guiding spatial navigation and supporting spatial memory.

### Targeted Stimulation of Hippocampal Place Cells Drives Spatial Behavior

We observed that stimulation of only a small fraction of the total place cell population can produce detectable effects on spatial navigation behavior, indicating that the representation is both efficient and sparse. Several mechanisms could potentially work in concert to explain this surprising result. The impact of our manipulation may have been aided by the observed place cell stimulation-driven inhibition of the endogenous place cell code, a result that supports the role of hippocampal interneurons in controlling network excitability and their potential contribution to spatial coding (Maglóczky and Freund, 2005; Wilent and Nitz, 2007; Grienberger et al., 2017). In addition, it is possible that the effects of our CA1 stimulation are amplified via attractor dynamics in downstream regions (Couey et al., 2013; Peyrache et al., 2015).

Earlier research involved the retrieval or manipulation of fear memories by controlling activity in hippocampal neurons with primarily context-specific but not spatial coding (Liu et al., 2012; Ramirez et al., 2013; Tanaka et al., 2018). While context-selective firing could support wider contextual memories, the parallel encoding of spatial location, or indeed the other finer modalities of an experience, may enable the formation of more detailed memories that allow navigation through the external world and within internal cognitive models.

### A Link Between Place Cell Activity and Spatial Learning

Our data suggest that animals underwent a degree of spatial learning as a result of driving Reward-PCs, evidenced by increased deceleration prior to the stimulation in later trials. Our stimulation will have influenced downstream neurons that play a role in guiding this spatial behavior. It is possible that these downstream neurons underwent potentiation with inputs from endogenously active stimulation zone place cells with firing fields that begin prior to the stimulation point. Activity in stimulation zone place cells may subsequently bias downstream reward-related activity, potentially supported by any reward-zone place cells that remap to become active before the stimulation point. The increased licking and deceleration we observed when driving Reward-PCs raises questions regarding how place cell activity influences downstream structures to guide behavior. One recent study illustrated the importance of the CA1 to nucleus accumbens projection for appetitive memory (Trouche et al., 2019), while future work should further explore the role of information flow through the subiculum (Kim et al., 2012), entorhinal cortex (Mizuseki et al., 2009a), and throughout the brain.

We observed an increased propensity for the animal to run too far past the reward zone when stimulating Start-PCs later in the track than their endogenous place fields. This result suggests that in some mice and trials, the task was being guided by a more internally generated path integration mechanism (Gallistel, 1990; Jayakumar et al., 2019) and that our stimulation was sufficient to shift this integrator to an earlier state, which was not then fully corrected to represent the current location by the track visual cues (Campbell et al., 2018). Future experiments on a longer virtual track and with different temporal patterns of stimulation should enable further exploration of this possibility.

One possible caveat with our reward-zone place cell stimulation experiments is that we cannot fully dissociate our target population from the potential small population of CA1 neurons that may encode reward location rather than pure spatial location (Gauthier and Tank, 2018). This population of neurons is unlikely to have contributed a significant proportion of our targeted Reward-PCs due to their rarity. Based on the proportion of reward coding neurons reported previously (Gauthier and Tank, 2018), the number of cells recorded in our task and the percentage of stimulation responsive cells, we estimate that on average only one or two reward coding neurons may have been activated during our Reward-PC stimulations. In addition, recent experiments report an increased field density of neurons encoding conjunctively the reward and the location rather than a purely reward location-specific sub-population (Lee et al., 2019). Future experiments will be required to fully dissociate these populations and assess their functional roles independently.

Another issue that must be considered is that viral delivery of the opsin construct under the CaMKII promoter may lead to non-specific expression in interneurons and astrocytes, which would allow their activity to be modulated by off-target light. To address this issue, we used immunohistochemistry to validate that C1V1 expression is predominantly restricted to pyramidal cells. Future experiments should leverage additional immunochemistry to more definitively rule out the possible expression of opsin in specific interneuron and astrocyte populations independently. While it is conceivable that a small fraction of interneurons did express low levels of C1V1, it is highly unlikely that this would affect our results: in addition to the observed lack of C1V1 expression in our GABA^+^ population, if off-target stimulation of interneurons were to contribute to the suppression of endogenous activity, we would expect to find the same suppression during the Non-PC control stimulation sessions. Despite Non-PC stimulation including more target neurons and higher overall light delivery to the tissue, this was not the case.

### Network-Level Place Cell Remapping Following Targeted Stimulation

We demonstrate that targeted optogenetic stimulation of place cells can lead to place cell remapping, extending the results of single-cell stimulation experiments in head-fixed (Bittner et al., 2015) and freely moving animals (Diamantaki et al., 2018). Importantly, our all-optical approach allows us to go beyond these results by demonstrating that remapping effects are not limited to the targeted neurons: indeed, we find widespread and persistent network-level remapping, with changes not being restricted to neurons that responded to stimulation or the subset of place cells that were targeted on that day. The mechanisms underlying these effects may include plasticity of excitatory synapses onto the stimulated cells (Bittner et al., 2015, 2017) as well as changes in the synaptic weight matrix underlying lateral inhibition in the CA1 network (English et al., 2017; McKenzie et al., 2019), supported by our observation of the inhibitory influence of stimulation on the endogenous place cell population. This in turn complements previous work showing that interneuron activity and pyramidal-interneuron functional connectivity are altered during spatial learning (Dupret et al., 2013) and that interneurons play a key role in regulating place cell firing (Royer et al., 2012) and place field formation (Sheffield et al., 2017; Turi et al., 2019). Investigating the contributions of the multitude of interneuron types throughout the hippocampus and their role in generating hippocampal maps promises to further our understanding of the neural mechanisms supporting memory and navigation.

### Outlook

Our results provide direct evidence for a causal role of hippocampal place cell firing in spatial cognition, thereby providing direct support for long-standing theories about the behavioral function of the hippocampal cognitive map (O’Keefe and Nadel, 1978; Eichenbaum et al., 1999; McNaughton et al., 2006). We also provide new insights into the network mechanisms underlying place field expression and population level remapping. Future studies are required to explore the importance of specific neural coding strategies in the hippocampus, particularly the role of temporal (O’Keefe and Recce, 1993; Skaggs et al., 1996; Feng et al., 2015) and rate coding (Huxter et al., 2003; Leutgeb et al., 2005), neural sequences (Skaggs and McNaughton, 1996; Lee and Wilson, 2002; Foster and Wilson, 2006; Diba and Buzsáki, 2007; Johnson and Redish, 2007; Foster and Wilson, 2007), the theta phase segregation of action potentials supporting memory encoding and retrieval (Hasselmo et al., 2002; Siegle and Wilson, 2014), and the readout of these activity patterns by downstream neurons and networks (Peyrache et al., 2009; Jadhav et al., 2016; Tingley and Buzsáki, 2018).

## STAR★Methods

### Key Resources Table

**Table:** 

REAGENT or RESOURCE	SOURCE	IDENTIFIER
**Antibodies**		

Rabbit polyclonal anti-GABA	Sigma-Aldrich	Cat# A2052; RRID: AB_477652
Goat anti rabbit IgG, Biotin SP conjugated	Merck-Millipore	Cat# AP132B; RRID: 11212148
Streptavidin conjugated Alexa Fluor 647	Thermo Scientific	Cat# S21374; RRID: 2336066

**Bacterial and Virus Strains**		

AAV-DJ-CaMKIIa-C1V1(E162T)-TS-p2A-mCherry-WPRE	Stanford Vector Core	GVVC-AAV-46
AAV1-Syn-GCaMP6f-WPRE-SV40	Addgene, Chen et al., 2013	100837-AAV9

**Chemicals, Peptides, and Recombinant Proteins**		

16% Formaldehyde (w/v), Methanol free (cat:28908)	Thermo Scientific	Cat# 28908
10X PBS	Thermo Scientific	Cat# AM9624
Tween 20	Sigma-Aldrich	Cat# P1379
Goat Serum	Merck-Millipore	Cat# S26-100ML

**Experimental Models: Organisms/Strains**		

Mouse: C57BL/6	Charles River Laboratories	Cat # 632

**Software and Algorithms**		

ImageJ	NIH	https://imagej.nih.gov/ij/
MATLAB	Mathworks	https://uk.mathworks.com/
Suite2p	Pachitariu et al., 2017	https://github.com/MouseLand/suite2p
Blender	The Blender Foundation	https://www.blender.org/
Virtual reality code in Python	https://github.com/neurodroid/gnoom	N/A

### Resource Availability

#### Lead Contact

Further information and requests for resources and reagents should be directed to and will be fulfilled by the Lead Contact, Michael Häusser (m.hausser@ucl.ac.uk).

#### Material Availability

This study did not generate new unique reagents.

#### Data and Code Availability

Data and analysis code are available from the authors upon request.

### Experimental Model and Subject Details

Wild-type C57BL/6 animals were obtained from Charles River Laboratories. Adult males between 14-18 weeks of age were used for all experiments. Animals were individually housed in an enriched environment within a temperature- and humidity-controlled, specific-pathogen free barrier facility at UCL. During experiments animals were water restricted to 85% of their body weight and food was available *ad libitum*. Animals were kept on a 12-hour light/dark cycle in a light reversal cupboard and experiments were performed during the dark epoch of the cycle. All animal procedures were approved by the local Animal Welfare and Ethical Review Board at University College London and performed under license from the UK Home Office in accordance with the Animals (Scientific Procedures) Act 1986.

### Method Details

#### Virtual reality and spatial navigation task

The virtual reality environment was a 200 cm linear track with various salient visual stimuli spanning its length (Figure 1C) created using Blender (Blender Foundation). Animals were head-fixed on a custom polystyrene wheel (12 cm wide, 20 cm diameter) with air bearings to minimize resistance. Running was tracked using a mouse optical sensor against the side of the wheel (Logitech G500) and was used to control the movement through the virtual world. The world was projected onto the inside of a large spherical dome (120 cm diameter) which covered the majority of the horizontal field of view of the mouse. The reward zone was defined as the area between 160 and 178.6 cm along the virtual track and the start zone was defined as the area between 21.4 and 48.8 cm. Animals were required to spend 3 s and lick 3 times in the reward zone to receive reward, while not licking > 10 times outside the reward zone or running too far into the back wall of the track. Licks were monitored using a custom-built electrical lick sensor. After reward delivery there was a minimum of 1 s delay and a requirement of 3 licks to end the trial and trigger the black time out inter trial interval. Failure according to either criteria resulted in instant teleportation to a white punishment time out environment. Successful trials were followed by a dark time out with a minimum of 5 s and unsuccessful trials were followed by a white time out with a minimum of 10 s. Animals were required to stop licking for at least 3 s in order to begin the next trial. On experimental days animals ran trials for a baseline period of 15 minutes. The data were analyzed and neurons were targeted, which took approximately 45 minutes. Then animals ran 5 minutes before stimulation, 10 minutes with stimulation, where every time the animal crossed the trigger point the same target population was stimulated, and 5 minutes post stimulation. Stimulation was triggered each time the animal crossed the 105 cm point of the virtual track and animals were allowed to run as many trials as they could complete in the epoch (19.2 ± 4.9 trials). On no stimulation control days animals ran trials for 15 minutes, before an hour delay period, followed by another 20 minutes of trials.

#### Behavioral training

Mice were initially placed onto the setup with no virtual reality projection for 15 minutes of acclimatization over 2 days. Subsequently initial training began with animals having to remain in the reward zone for 1 s, with no licks required to release the reward and no lick limit outside of the reward zone. Training sessions lasted ~30 minutes each day. Once animals were running well, we increased the time required in the reward zone to 2 s and then finally 3 s. When animals were stopping reliably, we added in a requirement for the animal to lick 3 times on target to receive reward, before finally adding a limit to the licks allowed outside of the reward zone, starting with 20 licks and working down to 10. Animals reached task performance in 17.4 ± 4.4 training sessions.

#### Virus injection, headplate installation and cannula implantation

Surgical procedures were similar to those described previously (Dombeck et al., 2010). All surgical procedures were performed under isoflurane anesthesia and analgesia (carprofen, buprenorphine). We first made a small (~0.5 mm diameter) craniotomy located over the right hippocampus, following which a mixture of AAV1-Syn-GCaMP6f-WPRE-SV40 (Chen et al., 2013, Addgene) and AAV-DJ-CaMKIIa-C1V1(E162T)-TS-p2A-mCherry-WPRE (Yizhar et al., 2011) (Stanford Gene Vector and Virus Core) was injected (1 μl at 100 nl/min) into dorsal CA1 (A/P −2.0 mm, M/L 1.5 mm, D/V −1.2 mm). About 2 weeks after virus injection, a second surgery was performed to install a custom-made headplate and implant a cannula with a window on the bottom (3 mm diameter, 1.6 mm height). After headplate installation, a 3 mm diameter craniotomy was made around the injection site using a biopsy punch. Cortical tissue within the craniotomy was slowly aspirated under repeated irrigation with cold saline until the external capsule was exposed. The cannula was inserted and cemented to the skull. After both surgeries, post-operative analgesics (carprofen) were administered for 3 days and mice were allowed to recover for at least 7 days.

#### Two-photon calcium imaging

Imaging was performed using a Thorlabs Bergamo II Rotating Multiphoton System with Spatial Light Modulator (Thorlabs) controlled using ThorImage (Thorlabs). A 930 nm laser beam from a Ti:sapphire laser (Chameleon Ultra II, Coherent) was focused onto the pyramidal layer of CA1 of the hippocampus at an average power of 60 mW through a 16x water-immersion objective (0.8 NA, Nikon). GCaMP6f fluorescence was amplified by photomultiplier tubes after being passed through a 562 nm long pass dichroic and a 525/50 nm bandpass filter. Images were acquired from a 800 × 800 μm field of view at a resolution of 512 × 512 pixels and a frame rate of 30 Hz. Light from the virtual reality projection was blocked from the objective to prevent contamination of the signal. Within experiment, videos were motion corrected and a correlation image was created where pixel values were weighted by correlation to neighboring pixels using functions adapted from Suite2P (Pachitariu et al., 2017). Neurons were selected as points of maxima in these images and manually curated. Fluorescence traces were extracted from circular regions of interest (ROIs) with a 5 μm radius around the selected centroid locations. Post experiment analysis was performed on calcium traces extracted using the Suite2P pipeline (Pachitariu et al., 2017). In brief, videos were motion corrected, neurons were detected, their calcium traces were extracted across time and these traces were corrected for any neuropil contamination. To ensure data quality and the stability of cells across our recording epochs we ran Suite2P separately on the baseline, stimulation and all epochs together (baseline, pre, stimulation and post). We manually curated the output of the baseline epoch, removing ROIs which did not correspond to cell soma or had insufficient signal. We then filtered for neurons which had a corresponding ROI throughout the session recordings (centroid location within 4 pixels) and selected these ROIs from the collectively processed data to ensure that we were looking at cells which were recorded stably and not lost to brain movement. On average 2.8 ± 3.4 target neurons did not pass our stability filter after each stimulation session. There was no difference in this number between session types (p = 0.9, Kruskal-Wallis test). In order to minimize this, following place cell analysis and targeting, any slight shift in the field of view was corrected if needed by matching it to an average image from the beginning of the baseline epoch. All analysis was performed using custom software written in MATLAB (MathWorks).

#### Place cell identification and classification

Place cells were identified from extracted calcium traces similarly to previous work (Dombeck et al., 2010; Danielson et al., 2016). Data from all trials were velocity filtered for periods when animals were running over 5 cm/s. For each ROI ΔF/F was calculated across time and drift in the baseline fluorescence was removed by normalizing by the 8th percentile value of a 10 s window around the imaging frame. For each virtual reality trial the ΔF/F value for each ROI was extracted as a function of virtual space and the average ΔF/F over space was calculated for 2.27 cm spatial bins and smoothed with a Gaussian kernel with an SD of 3 bins. Place field criteria were then applied to these traces as reported previously (Dombeck et al., 2010), but without the bootstrapping measure to minimize the time between baseline and stimulation and with a minimum field peak value of 20% ΔF/F to reduce the occurrence of any false positives. Start zone place cells were classified as those with fields which overlapped with > 50% of the 21.4 – 48.8 cm area of the track. Reward zone place cells were classified as those with fields which overlapped with > 50% of the 160 - 178.6 cm rewarded area of the track. The start zone definition was larger to allow better matching of the populations targeted for stimulation. The bounds of these zones were equidistant from the stimulation trigger point of the virtual track. Non-place cells were selected as neurons with clear full soma in the correlation image generated from the baseline epoch recording and which did not pass the spatial tuning criteria of our place cell analysis. We compared activity rates between these neurons and place cells, both in and out of the VR world, by calculating the number of times the ΔF/F trace reached 3 SD above the mean value during each epoch and then converting this to a rate.

#### Two-photon holographic optogenetic stimulation

Simultaneous all-optical two-photon imaging and two-photon optogenetic stimulation was carried out similarly to as previously described (Packer et al. 2015; Carrillo-Reid et al., 2016; Yang et al., 2018; Marshel et al., 2019). On a given experimental day either the start zone place cells, reward zone place cells or non-place cells were stimulated upon crossing the 105 cm point of the virtual track. Neurons were targeted using their centroid locations in ThorImage (Thorlabs). A second light path on the microscope utilized a 1030 nm femtosecond fiber laser (BlueCut 10, Menlo Systems) and a reflective multilevel spatial light modulator (SLM; OverDrive Plus SLM, Meadowlark Optics/Boulder Nonlinear Systems; 7.68 × 7.68 mm active area, 512 × 512 pixels, optimized for 1064 nm). The Gerchberg-Saxton algorithm (Gerchberg and Saxton, 1972) was used to calculate holograms to be displayed on the SLM to shape beamlets corresponding to the cluster of neurons to be targeted, allowing simultaneous stimulation of that population. Imaging and stimulation lasers were combined at a 740 nm short pass and a 1050/40 nm band pass custom dichroic (Thorlabs). In order to target as many neurons from one experimental group as possible we clustered the place cell targets into 5 groups across our large imaging field of view and used a galvo-galvo scanner (Thorlabs) to direct our stimulation onto each cluster sequentially while changing the phase mask on the SLM to provide the required beamlets. Neurons were targeted with an average power of 6 mW per cell, which was spiraled over an 8 μm diameter area in 10 ms and 10 times in succession for 100 ms of stimulation onto each cluster. A 5 ms gap between stimulation to clusters allowed SLM phase mask switching and galvo-galvo scanner movement. The 5 clusters were cycled through twice for a total of 1000 ms of stimulation over 1045 ms.

We quantified the optical resolution of our stimulation pathway by taking a Z-stack of a 1 μm fluorescent bead using a single stimulation beamlet and measuring the signal along each axis at different distances from its peak intensity. We generated physiological resolution curves by targeting light at different displacements (as in Marshel et al., 2019, over a range of ± 30 μm for X/Y and ± 55 μm for Z) from the soma of C1V1 and GCaMP6f expressing neurons and then stimulating for 30 trials using the same protocol as our experimental data and with an 10 s delay between trials. We then extracted the average ΔF/F value for each neuron over a 100 ms window after the stimulation and normalized this by the maximum across all stimulation displacements. We targeted clusters of cells simultaneously to make the stimulation closely comparable to that used during experiments.

#### Immunohistochemistry

For tissue preparation animals (n = 3) underwent terminal anesthesia and transcardial perfusion fixation with 4% paraformaldehyde in 0.1 M phosphate buffer (PBS; pH 7.2). Dissected brains were kept immersion fixed overnight at 4°C. The next day 60 μm coronal full brain sections were cut on a vibratome and rinsed in PBS. Sections were selected from the region under the cranial window / cannula, where both mCherry and GFP signals were present. Slices were then rinsed in PBS, followed by pre-incubation with 10% goat serum (NGS) in 0.1 M PBS with 0.5% Tween 20 (PBS-T, Sigma Aldrich) for 2 hours. Immunopositivity for GABA was tested with a primary antibody raised in rabbit (1:100, Sigma Aldrich, rabbit, polyclonal). Slices were kept in primary antibody solution with 3% NGS in PBS-T on a shaker overnight in a cold room, followed by PBS washing the next day. To enhance the signal from the primary antibody, slices were incubated in biotin SP conjugated goat anti rabbit IgG (1:500, Millipore, goat, polyclonal) for 2 hours at room temperature. After extensive washing with PBS to visualize the immunoreaction streptavidin AF 647 (1:250, Thermo Fisher) was used for another 2 hours at room temperature. Following final rinsing and mounting fluorescent images were acquired with a Zeiss LSM 880 Airyscan confocal microscope, using 20x and 63x objectives.

To quantify the immunohistochemical staining, ROIs were drawn over putative interneurons which were GABA^+^ and in or immediately adjacent to the CA1 pyramidal layer. We excluded putative astrocytes based on visual morphological identification from confocal images. Putative astrocytes were primarily identified by their distinctive fusiform cell body, exhibiting multiple radiating and tapering primary processes that branched into many fine smooth processes (Nixdorf-Bergweiler et al., 1994; Bushong et al., 2002; Hama et al., 2004). Note that not excluding putative astrocytes did not alter the results of our immunohistochemistry quantification. For each ROI we then took the mean fluorescence value from both the GABA and the C1V1 associated channels. For each GABA^+^ ROI we also drew an ROI around a GABA^-^ neuron from within the pyramidal layer of the same image, before taking the same values. In addition to these measurements we placed a ROI over an area outside the pyramidal cell layer where no clear neurons were present. For each GABA^+^ and GABA^-^ putative neuron we then subtracted this background signal level to obtain background-corrected values.

### Quantification and Statistical Analysis

#### Response analysis

To identify neurons which responded to our photostimulation the fluorescence trace for each neuron was extracted for each trial for 30 frames before and 120 frames after the bout of stimulation was triggered. These traces were then normalized by subtracting and dividing by the mean baseline fluorescence value from frames 5-29 of the stimulus triggered trace for that neuron on each trial. The artifact from the stimulation laser was removed from imaging frames captured during stimulation using methodology described previously and adapted for our imaging and stimulation parameters (Yang et al., 2018). Cell responses due to stimulation were considered in windows corresponding to ~100 ms after each neural cluster stimulation was completed, resulting in 10 stimulation windows. The preferred stimulation window for each cell was chosen based on the maximum trial-averaged response. We defined responsive cells as those with GCaMP6f signal reaching ΔF/F > 40% on > 30% of trials in their preferred stimulation window. To identify neurons that were stimulated off-target we applied this method to all neurons in each recording. To prevent the inclusion of false positives due to tuned endogenous activity, remapping or imperfect artifact subtraction, we removed cells which passed the same response analysis during no stimulation epochs and those that were not proximal enough to a laser beamlet to have been indirectly stimulated. We used the conservative limit of 30 μm, based on our optical and physiological resolution measures (Figures S1I and S1K). To assess response stability the average response magnitude of the responding target population was calculated across trials for each stimulation epoch and a linear fit was made. The slopes were then compared to zero and no detectable difference was found (Figure S2D).

#### Behavioral analysis

Position, running speed, and event codes such as licks, reward delivery and trial starts were captured by our virtual reality software at a 100 Hz sample rate. The virtual linear track was binned into 3.03 cm bins and for each trial the lick rate, speed, occupancy and deceleration events were calculated across space. Acceleration was calculated across 0.1 s intervals and deceleration events were defined as an acceleration 2 standard deviations below the mean. Good trials were defined as those where the animal reached the stimulation point (105 cm) and were used for analysis involving the stimulation data. For session-wise measures the distribution of each metric across space was averaged across trials, for within-animal measures these sessions were then averaged together and for total average measures each animal’s average curve was then averaged together to give each animal equal weight. For all stimulation delta measures the distribution of the variable across space during the baseline epoch was subtracted from the same distribution from the stimulation epoch. For pre-post behavioral changes the pre epoch distribution was subtracted from the post epochs. Summary statistics were run on the mean value within defined spatial windows. These windows covered the 21.21 cm after the stimulation trigger or over the reward zone for the lick rate and 15.15 cm prior to the stimulation trigger for the deceleration events comparison. These were chosen to fit the amount of space covered by slower animals during stimulation or the average amount of space prior to the stimulation point that animals began to decelerate prior to the reward zone in baseline epochs, taken as the distance to half the peak in events. For trial outcome comparisons the difference in the proportion of a certain trial outcome between the baseline and the stimulation epochs was calculated and the same value from no stimulation control days was then subtracted for that mouse to take into account any non-stimulation related changes in trial outcome over the one hour delay. To look more closely at the relationship between behavioral change and the specificity and efficacy of place cell activation we calculated the specificity of the stimulation to one place cell group, defined as the number of responding reward zone place cells minus the number of responding start zone place cells, divided by the total number of responding neurons. We then multiplied this specificity by the total number of responsive cells and the average ΔF/F magnitude of the response.

#### Network activity factor analysis

After removal of imaging frames acquired during stimulation, ΔF/F traces were z-scored within each epoch and spatially binned. Traces were concatenated across all trials in a session to form a data matrix of cell activity. Factor analysis was performed using the MATLAB function *factoran* (10 factors), with the regression method for estimating factor scores (MathWorks). Changes in network activity due to stimulation were quantified by the Euclidean distance in latent space between the trial-averaged trajectories from stimulation and control (Pre) epochs of each session (Rickgauer et al., 2014). Within stimulation conditions, Euclidean distances at each spatial bin following the stimulation zone were compared to averages over the ten spatial bins preceding the stimulation zone using Wilcoxon sign-rank tests. The associated perturbations in place cell activity were computed as the difference in trial-averaged cell activity between the stimulation and pre epochs, averaged over three spatial bins following the stimulation zone (centered at 136 cm; selected such that the majority of sessions were included after removal of stimulation affected imaging frames). Cells with factor loadings less than 0.2 for all latent factors, or with average difference in activity of more than 0.2 standard deviations over the ten spatial bins preceding the stimulation zone were excluded. We confirmed that our results were robust to the exact values of these thresholds. Suppressed cells were defined as those whose difference in activity was below the 10th percentile for a given session, and enhanced cells as those whose difference in activity was above the 90th percentile. The same cell selection procedure was applied to the no-stimulation condition, with epochs defined by experimental time, to form null distributions for statistical comparison. Stimulus-triggered averages of example cells in Figures 4C and 4D were computed from the standardized ΔF/F signal, aligned to the time of stimulus onset and smoothed with a Gaussian filter (SD of 5 imaging frames) before averaging.

#### Remapping analysis

To characterize lasting changes in spatial representations following optogenetic stimulation, we compared place fields between pre- and post-stimulation epochs. Cells that did not appear stable (Pearson correlation coefficient of < 0.3 between trial-averaged place field maps) across baseline and pre-stimulation epochs were excluded from further analyses. To assess the similarity of spatial representations before and after stimulation, we compared Pearson correlation coefficients between trial-averaged place field maps of pre- and post-stimulation epochs (Muller and Kubie, 1987; Bostock et al., 1991). The position of a cell’s place field was quantified as the trial-averaged center of mass (COM) of the neuron’s calcium activity during the pre- or post-stimulation epoch, respectively. The peak of a place field COM distribution was defined as the mode of a normal kernel fit to the COM histogram. For the place cell peak shuffled distribution all cells average calcium traces across space were randomly translocated along the track, before counting the number of peaks in the reward zone.
