# Targeted Activation of Hippocampal Place Cells Drives Memory-Guided Spatial Behavior

**DOI:** 10.1016/j.cell.2020.12.010

**Published:** 2020-12-23

**Authors:** Nick T.M. Robinson, Lucie A.L. Descamps, Lloyd E. Russell, Moritz O. Buchholz, Brendan A. Bicknell, Georgy K. Antonov, Joanna Y.N. Lau, Rebecca Nutbrown, Christoph Schmidt-Hieber, Michael Häusser

(Cell *183*, 1586–1599.e1–e10; December 10, 2020)

After online publication we discovered presentational errors in two figures. The colors in the lower two plots of Figure 5E did not correspond correctly to the data labels, and the y axis tick labels of Figure S3B were displayed in m s^-1^ rather than cm s^-1^. These mistakes have been corrected, and did not affect the data analysis, results or conclusions. We apologize for any inconvenience caused.

**Figure 5:**
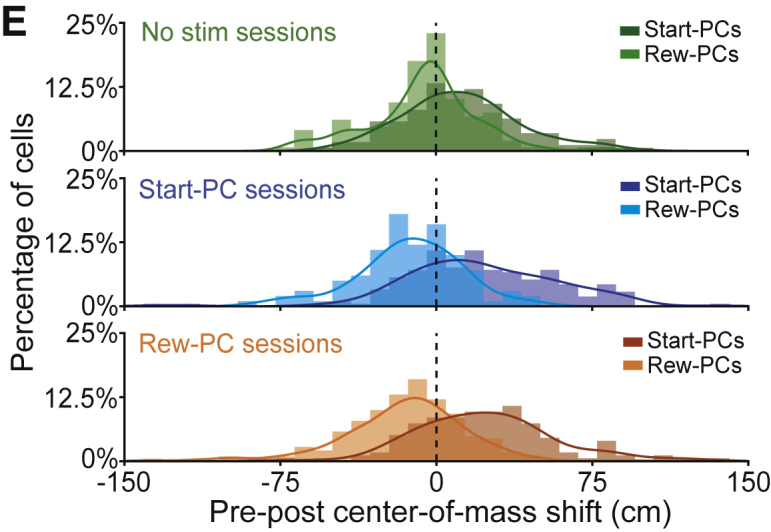
Stimulation-Driven Remapping Influences Spatial Behavior (corrected)

**Figure 5:**
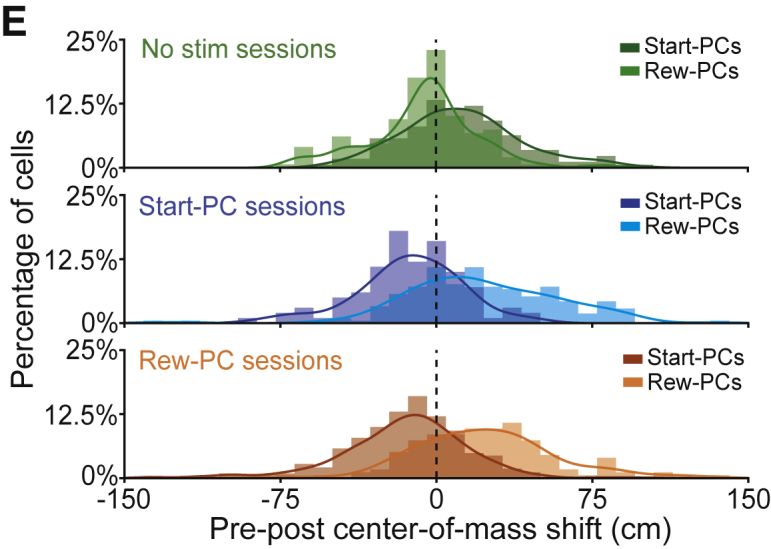
Stimulation-Driven Remapping Influences Spatial Behavior (original)

**Figure S3:**
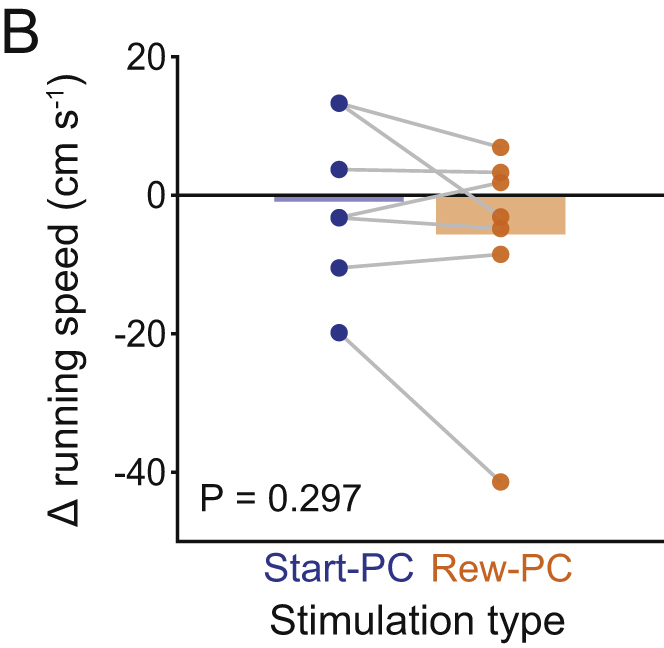
Effect of Stimulation on Running Speed (corrected)

**Figure S3:**
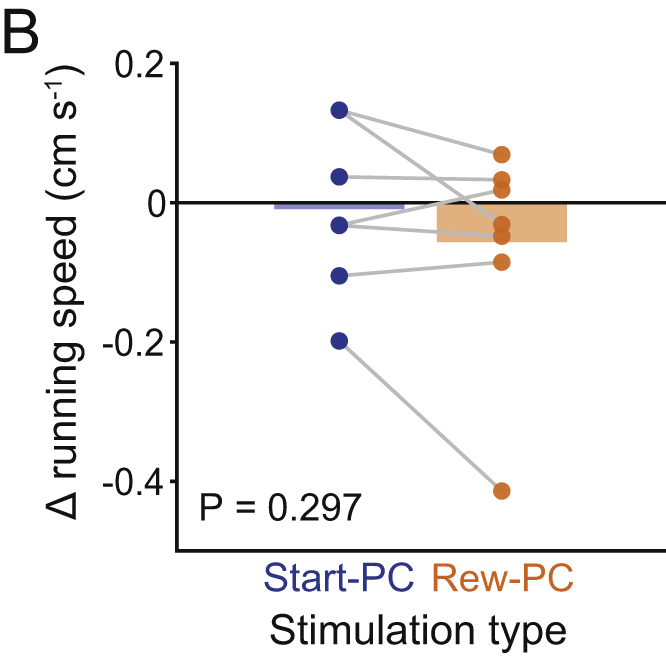
Effect of Stimulation on Running Speed (original)

